# Genomic and functional divergence analysis of closely related *Teseptimavirus* phages reveals dual HNH endonuclease positioning and its role in plaque formation

**DOI:** 10.1128/aem.01347-26

**Published:** 2026-09-23

**Authors:** Xinyi Yang, Siying Zheng, Bei Zhou, Shuya Li, Hauke Smidt, Weiyun Zhu, Yan Lin

**Affiliations:** 1 Laboratory of Gastrointestinal Microbiology, Jiangsu Key Laboratory of Gastrointestinal Nutrition and Animal Health, College of Animal Science and Technology, Nanjing Agricultural University 524556, Nanjing, China; 2 National Center for International Research on Animal Gut Nutrition, Nanjing Agricultural University 610933, Nanjing, China; 3 Laboratory of Microbiology, Wageningen University & Research 568404, Wageningen, the Netherlands; University of Nebraska-Lincoln, Lincoln, Nebraska, USA

**Keywords:** polyvalent bacteriophage, *Teseptimavirus*, efficiency of plating, genome comparative analysis, HNH endonuclease

## Abstract

Antibiotic resistance has renewed interest in bacteriophages as antimicrobials, yet the complexity of phage-host interactions often leads to inconsistent infection outcomes, underscoring the need to unravel the molecular basis of phenotypic diversity among closely related phages. Here, we compared two closely related T7-like phages, polyvalent phages C5 and N30, both belonging to *Teseptimavirus*, which share 89.67% genomic identity and an identical host range. While C5 exhibited a higher adsorption rate, plaque size, and efficiency of plating, N30 showed stronger bacteriostatic activity and larger burst sizes, highlighting the importance of a comprehensive evaluation of phage infection traits for therapeutic or biocontrol applications. Comparative genomics revealed high overall similarity between phages C5 and N30 but notable divergence in a hypothetical protein and a histidine-asparagine-histidine (HNH) endonuclease. Broader analysis across *Teseptimavirus* further uncovered two distinct genomic positions of the HNH endonuclease: either adjacent to virion assembly proteins in the genome middle or next to the terminase large subunit at the genome end, suggesting functional specialization in capsid assembly and DNA packaging. Tertiary structure prediction and comparison of the HNH endonucleases revealed moderate structural similarity despite moderate sequence divergence. Furthermore, a single nucleotide substitution in the HNH gene of N30 markedly increased its efficiency of plating in one tested host strain, demonstrating that terminase-associated HNH endonuclease plays a pivotal role in plaque formation. Together, these findings provide the first evidence of dual HNH endonuclease positioning within *Teseptimavirus* and identify the HNH endonuclease as a key genetic determinant of plaque formation, offering new insights into the molecular basis of phage infection variability and host adaptation.

**IMPORTANCE:**

Closely related bacteriophages often display strikingly different infection phenotypes, yet the genetic basis of this variation remains poorly understood. Here, we uncover a previously unrecognized feature within the genus *Teseptimavirus*: the HNH endonuclease can occupy two distinct genomic positions, either associated with virion assembly or DNA packaging modules, suggesting functional specialization. We further demonstrate that this enzyme is closely linked to infection phenotypes, as even a single nucleotide substitution in the HNH gene significantly alters the efficiency of plating. These findings identify the HNH endonuclease as a key determinant of phage infectivity and reveal that its genomic context may shape functional outcomes. This work provides new insight into how subtle genetic differences drive phenotypic divergence among closely related phages and highlights the HNH endonuclease as a promising target for rational phage optimization in therapeutic and biocontrol applications.

## INTRODUCTION

Bacteriophages (phages) are the most abundant biological entities in the biosphere and are key drivers of bacterial mortality and population turnover across diverse ecosystems. The escalating global crisis of antibiotic resistance has reignited interest in phage therapy as a promising alternative antimicrobial strategy (1, 2). Unlike broad-spectrum antibiotics, phages infect bacteria through highly specific phage-host interactions, and the two engage in a continuous ‌evolutionary arms race‌ (3). This ongoing coevolution frequently gives rise to therapeutic challenges, including alterations in host range and variations in infection efficiency. However, even closely related phages often display strikingly different infection outcomes on the same bacterial host (4), reflecting the complexity of phage-host interactions and limiting the predictability of phage therapy. Therefore, elucidating the molecular mechanisms underlying these phenotypes is essential for improving the predictability and robustness of phage applications.

Phage infection is a multistep process involving host adsorption, genome injection, intracellular replication, virion assembly, and cell lysis. Variations at any stage can lead to measurable differences in infection phenotypes. Several parameters are commonly used to characterize phage infection, including adsorption rate, efficiency of plating (EOP), latent period, burst size, and bactericidal activity, each reflecting different stages of the infection cycle (5–10). Among these, the EOP is particularly informative because it integrates the overall success of productive infection, encompassing successful adsorption, intracellular replication, progeny assembly, and release to generate visible plaques on a bacterial lawn. Consequently, EOP is widely used to evaluate phage infectivity, host susceptibility, host range, and adaptation to different bacterial strains. However, despite its broad application, the genetic basis underlying EOP variation remains poorly understood. This research gap is largely attributable to the limited functional annotation of phage genomes. Although phage genes and proteins play essential roles at every stage of infection (11), the vast majority of phage genomes remain uncharacterized (12), and the functions of many predicted genes are still unknown. As a result, the contributions of specific phage genes to individual infection processes remain largely unresolved.

We previously isolated two polyvalent phages, C5 and N30, infecting *Escherichia coli* (*E. coli*) and *Citrobacter freundii* (*C. freundii*) strains from the pig gut (4). Despite sharing 89.67% genomic similarity and exhibiting identical host ranges across five tested bacterial strains, C5 consistently displayed higher EOP than N30. This phenotypic divergence among closely related phages provides an opportunity to investigate the genetic determinants underlying variation in infection efficiency. In this study, we further compared key infection phenotypes of C5 and N30, including plaque morphology, adsorption efficiency, phage, and bacterial growth dynamics, and performed comparative genomic analyses to identify candidate genes potentially associated with differences in infectivity. These findings provide new insights into the genetic basis of phage-host interactions and may facilitate the rational engineering of phages with improved infection performance.

## RESULTS

### Comparison of infection characteristics between phages C5 and N30

**Fig 1:**
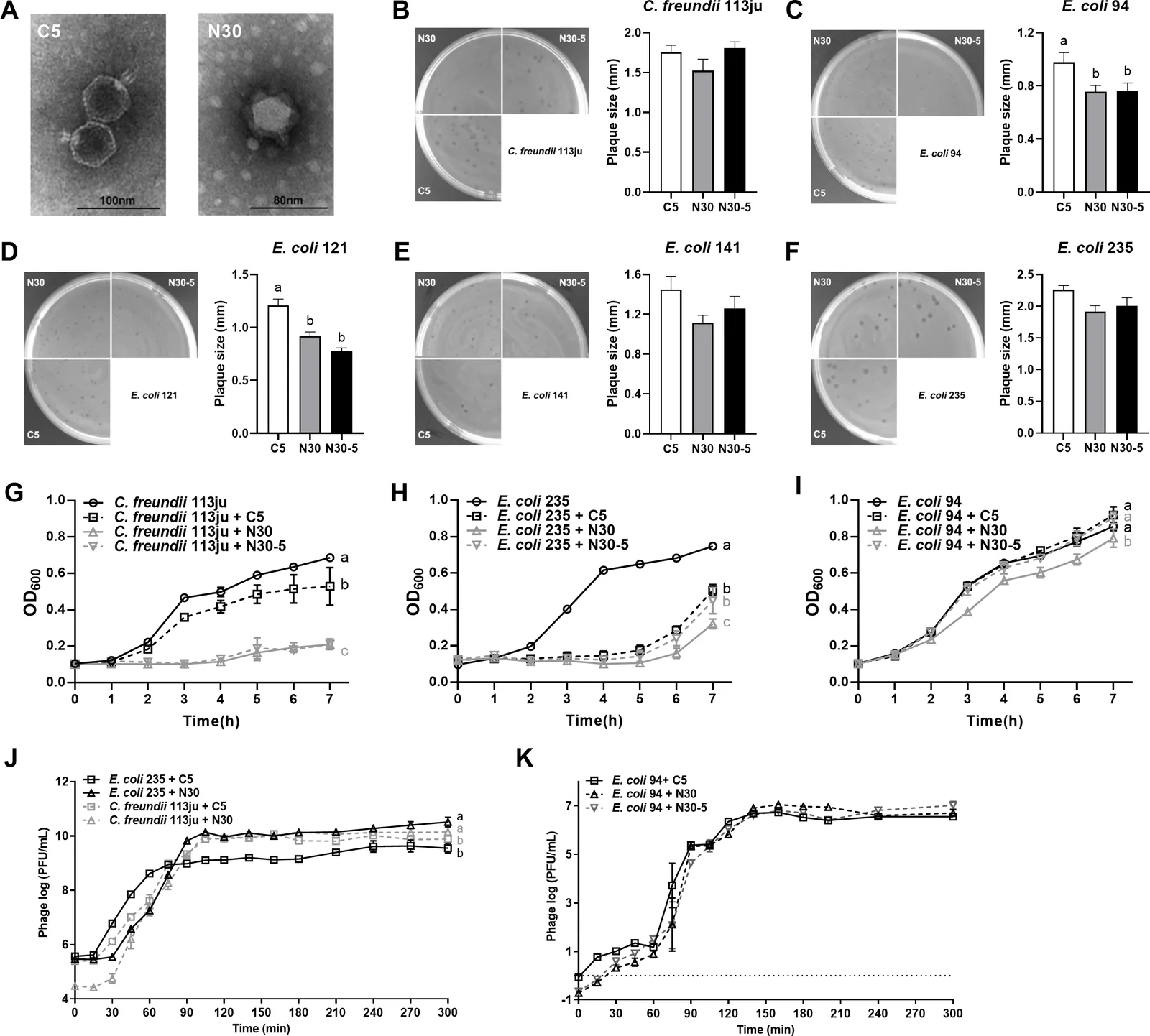
Morphological and infection characteristics of phages. (**A**) Transmission electron microscope images of C5 and N30. Representative plaque morphologies and diameters of C5, N30, and N30-5 on *C. freundii* 113ju (**B**), *E. coli* 94 (**C**), *E. coli* 121 (**D**), *E. coli* 141 (**E**), and *E. coli* 235 (**F**). Panel labels are self-explanatory. *C. freundii* 113ju (**G**), *E. coli* 235 (**H**), and *E. coli* 94 (**I**) growth curves in the presence of phages. One-step growth curves of C5, N30, and N30-5 on *E. coli* 235, *C. freundii* 113ju (**J**), and *E. coli* 94 (**K**). Error bars indicate the standard error of the mean. Different small letters denote statistically significant differences between groups at *P* < 0.05.

Polyvalent phages C5 and N30 were previously reported to share the same host range of infection, including *C. freundii* 113ju, *E. coli* 94, *E. coli* 121, *E. coli* 141, and *E. coli* 235, but the EOPs of C5 on these five strains were significantly higher compared to those of N30 (4). In this study, we aim to characterize the infection phenotypes of these two phages and explore the possible molecular basis underlying their differences. Transmission electron microscopy revealed that both C5 and N30 exhibited typical *Podovirus* morphology, characterized by an icosahedral capsid and a short, non-contractile tail (Fig. 1). We compared the plaque size, adsorption rate, and EOP of phages C5 and N30 across five bacteria to systematically evaluate key infection-related phenotypes and compare infection characteristics between the two phages. C5 consistently produced significantly larger plaques than N30 on two of the five tested bacteria (Fig. 1B through F), suggesting more efficient plaque development and spread on bacterial lawns. In addition, C5 exhibited significantly higher adsorption rates on three of the five hosts and higher EOP values across all five bacteria, indicating enhanced host attachment efficiency and a greater capacity to establish productive infections (Table 1). To investigate the infection dynamics of C5 and N30, subsequent analyses were performed using three representative hosts. In addition to *C. freundii* 113ju, the original isolation host, we selected *E. coli* 235 and *E. coli* 94, which represented the most susceptible and least susceptible hosts, respectively, based on their plaque size, adsorption rate, and EOP profiles. This host selection allowed us to capture a wide range of phage-host interaction outcomes while minimizing redundancy among hosts with similar susceptibility patterns. Bacteriostatic assays were subsequently performed using these three bacteria. Unexpectedly, N30 displayed markedly stronger inhibitory activity than C5 against *C. freundii* 113ju (Fig. 1G), *E. coli* 235 (Fig. 1H), and *E. coli* 94 (Fig. 1I). Consistent with these findings, one-step growth curves demonstrated that N30 produced a significantly larger burst size than C5 in *C. freundii* 113ju and *E. coli* 235 and reached a higher final phage titer in *E. coli* 235 but no significant difference in *E. coli* 94 (Fig. 1J and K).

**TABLE 1:** Adsorption rate and efficiency of plating of phages C5, N30, and N30-5 in five bacteria^*a*^

Phage	*C. freundii* 113ju	*E. coli* 94	*E. coli* 121	*E. coli* 141	*E. coli* 235
Adsorption rate					
C5	0.16 ± 0.044^a^	0.051 ± 0.0014^a^	0.00207 ± 0.00060^a^	0.083 ± 0.023	0.23 ± 0.03
N30	0.043 ± 0.011^b^	0.00056 ± 0.00010^b^	0.00068 ± 0.00016^b^	0.050 ± 0.0086	0.26 ± 0.04
N30-5	0.045 ± 0.012^b^	0.00058 ± 0.000089^b^	0.00043 ± 0.000067^b^	0.053 ± 0.017	0.23 ± 0.067
*P* value	0.013	0.002	0.016	0.349	0.863
Efficiency of plating					
C5	1	0.75 ± 0.078^a^	1.15 ± 0.17^a^	1.16 ± 0.17^a^	1.77 ± 0.16^a^
N30	1	0.0037 ± 0.0011^c^	0.35 ± 0.044^b^	0.56 ± 0.095^b^	0.55 ± 0.11^b^
N30-5	1	0.041 ± 0.013^b^	0.42 ± 0.042^b^	0.56 ± 0.12^b^	0.86 ± 0.079^b^
*P* value		<0.001	<0.001	0.011	<0.001

^*a*^ Data are expressed as arithmetic means ± standard deviations. Different letters denote statistically significant differences among phages within the same bacteria at *P *< 0.05.

### Comparative genomics and protein sequence alignment of phages C5 and N30

**Fig 2:**
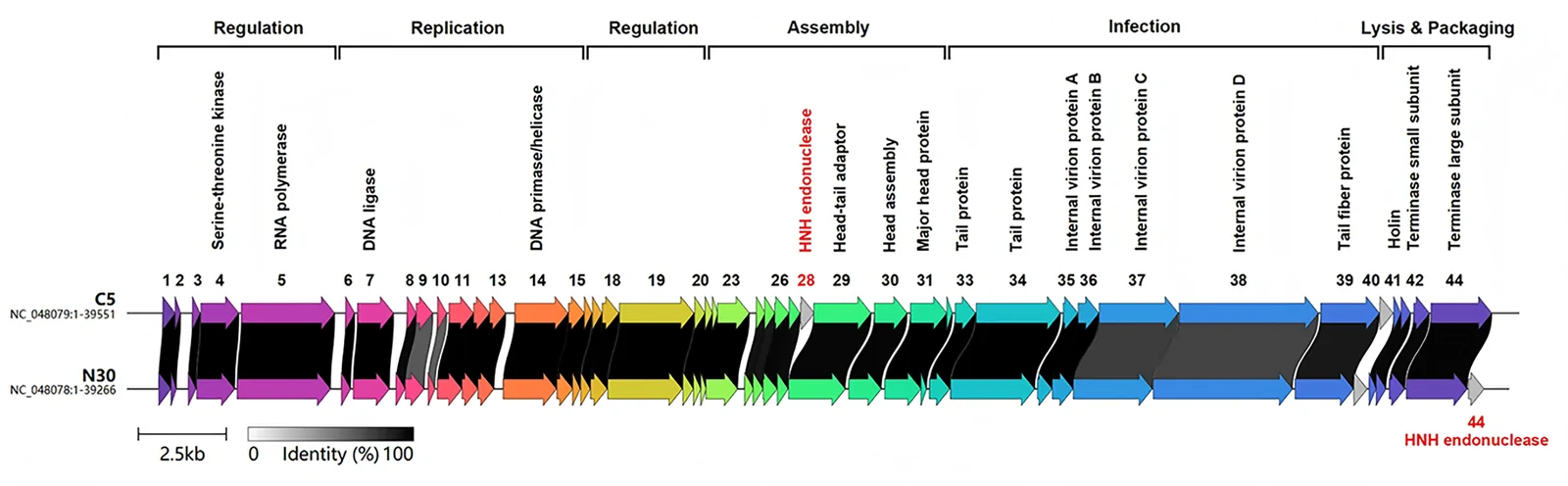
Genome alignment and protein identity comparison between phages C5 and N30. The linear map is based on nucleotide sequences of the phage genomes and predicted ORFs. The ORFs are numbered, and selected genes are annotated. Each arrow represents a predicted ORF, with arrow orientation indicating the transcriptional direction. The grey-scale bars connecting the two genomes indicate amino acid sequence identity between homologous ORFs, where darker shading corresponds to higher identity.

C5 and N30 both possess linear double-stranded DNA genomes, with sizes of 39,551 and 39,266 bp, respectively, showing approximately 92% genetic identity to phage T7 (39,937 bp), and both belong to the *Teseptimavirus* within the subfamily *Studiervirinae*. The GC content of C5 and N30 genomes is 48.82% and 48.61%, respectively, reflecting the high degree of sequence identity. The length of the direct terminal repeat in the N30 genome was identical to that observed in T7 (160 bp), whereas C5 exhibited a repeat sequence length of 134 bp. Pairwise comparison of the proteins predicted to be encoded by C5 and N30 genomes revealed that both phages encode 44 open reading frames (ORFs) (Fig. 2), with the exception of N30_ORF44 and C5_ORF28, which could not be paired due to low sequence identity and their distinct genomic positions. All other ORFs of the two phages shared the same genomic arrangement. Among the paired proteins, 33 shared greater than 95% amino acid identity, 25 of which were identical. An additional four proteins, including the tail fiber protein and regulatory proteins, showed 90%–95% identity. Furthermore, three proteins exhibited 70%–90% identity, including nucleotide kinase, internal virion protein C, and internal virion protein D. C5_ORF28 and N30_ORF44 were the only ORFs that could not be paired, sharing only 46.25% protein sequence identity. BLASTp analysis against phage T7 revealed that C5_ORF28 matched T7_gp7.7 (annotated as a hypothetical protein but containing an HNH endonuclease domain) with 100% coverage and 71.54% identity, while N30_ORF44 showed only 61% coverage and 45% identity to its best match. The top matches for both proteins were exclusively from phages within the *Teseptimavirus*. Pfam domain analysis further showed that both proteins contain an HNH endonuclease domain, which is located within the N-terminal 56% of their primary sequences.

**Fig 3:**
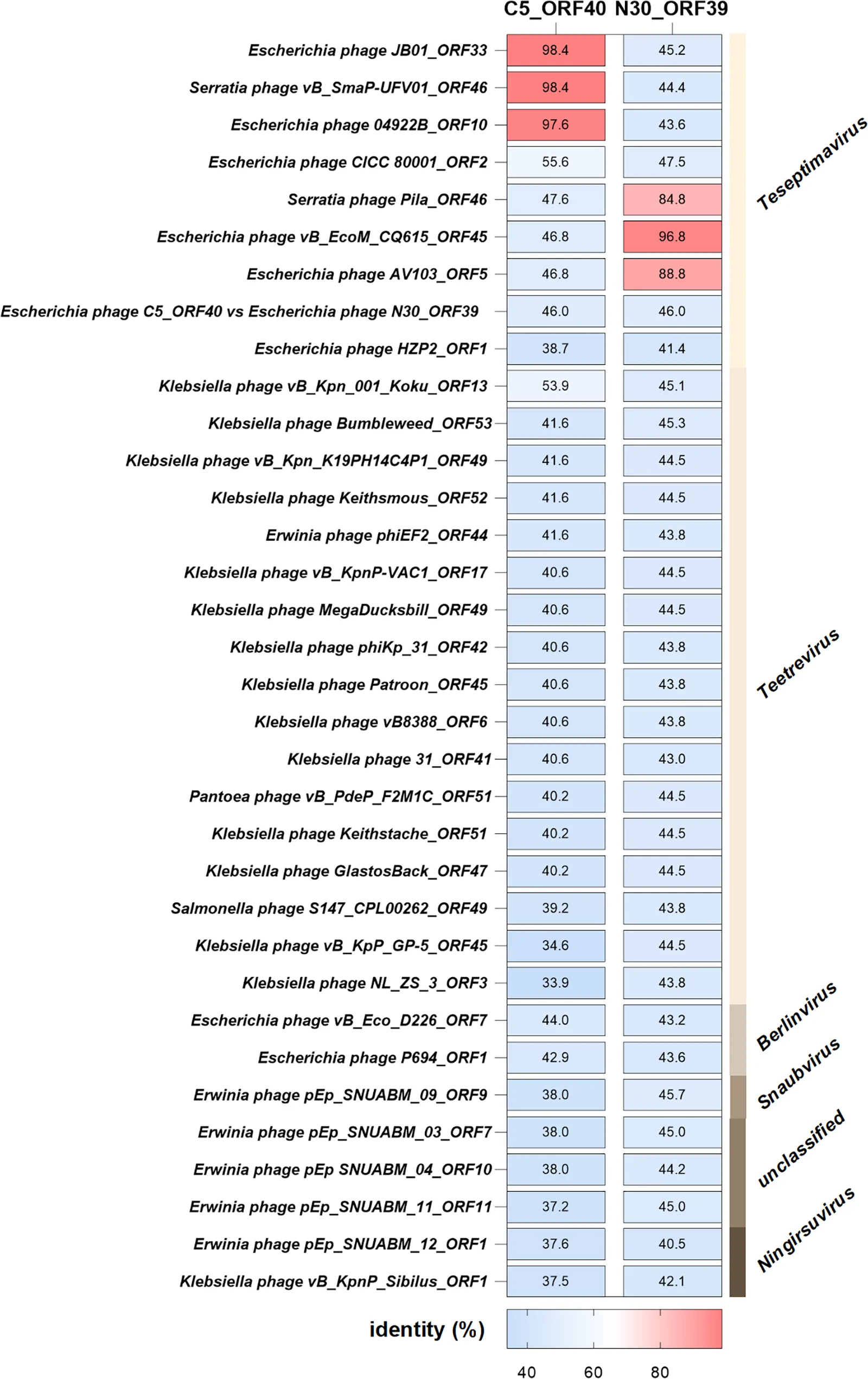
Heatmaps of amino acid identity between C5_ORF40 and N30_ORF39 and their best BLASTp hits against the NR database. The *y*-axis represents C5_ORF40 and N30_ORF39, while the *x-*axis denotes representative sequences from different phage genera within the subfamily *Studiervirinae*. The color intensity of each cell reflects the amino acid identity between the corresponding ORF and its best match.

Another paired ORF with relatively low sequence identity (46%) was C5_ORF40 and N30_ORF39. Notably, BLASTp searches identified only five and four proteins sharing >50% identity with C5_ORF40 and N30_ORF39, respectively. Of these nine proteins, one belongs to a bacterium, eight belong to *Teseptimavirus* phages, and none have been functionally characterized. Interestingly, all proteins with low similarity to N30_ORF39 and C5_ORF40 were exclusively derived from phages within the subfamily *Studiervirinae* (Fig. 3). However, these proteins are not universally conserved across all *Studiervirinae* phages, and even considerable sequence variation exists even among members of the same genus. Such pronounced sequence plasticity suggests that these proteins represent dynamic genetic elements undergoing active remodeling across phage lineages. Notably, when the comparison was restricted to genera other than *Teseptimavirus*, C5_ORF40 exhibited a more heterogeneous similarity landscape across genera (37.2%–53.9%), whereas homologs of N30_ORF39 appeared more uniformly distributed (40.5%–45.7%). More than half of the homologous sequences identified for C5_ORF40 and N30_ORF39 belonged to the genus *Teetrevirus*. Despite detectable sequence similarity, all identified homologs were annotated as hypothetical proteins, with no functional assignments available in public databases.

### Identification of the mutation causing significant changes in EOP of phages

**Fig 4:**
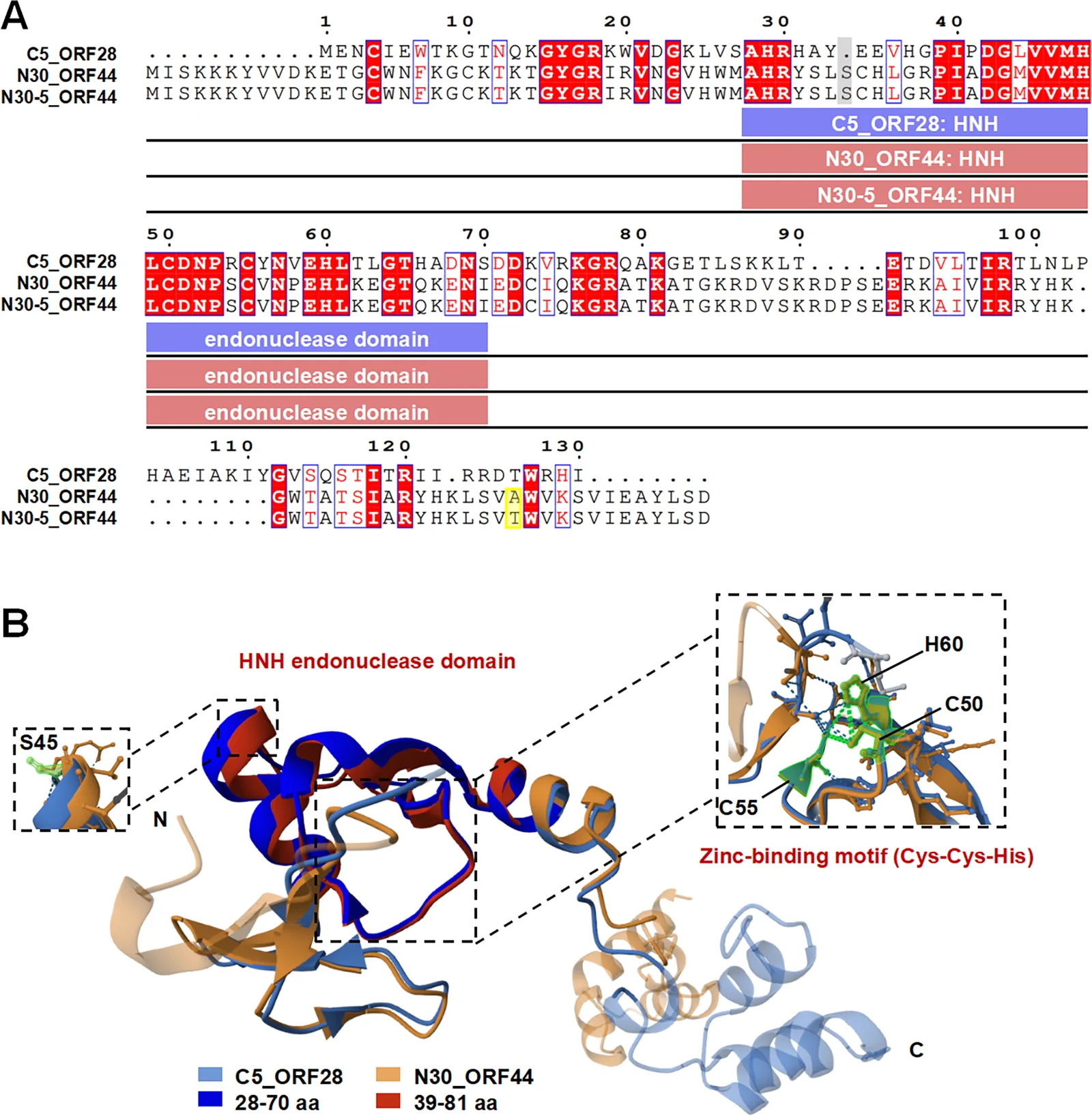
Multiple sequence and three-dimensional structural comparison of ORFs containing HNH endonuclease domain. (**A**) Sequence alignment of C5_ORF28, N30_ORF44, and N30-5_ORF44. Red boxes denote conserved residues. Blue frames indicate positions with high physicochemical similarity within the same amino acid group. The gray boxes mark the extra residue at S45 in N30_ORF44 compared to C5_ORF28. The yellow boxes mark the single amino acid difference between N30_ORF44 and N30-5_ORF44. (**B**) Three-dimensional structure predictions of C5_ORF28 and N30_ORF44. The predicted catalytic residues in C5_ORF28 (C50, C55, and H60) and the extra residue at S45 in N30_ORF44 are highlighted in green.

Given the striking difference in EOP between C5 and N30 on *E. coli* 94, with N30 plating extremely poorly, this strain was chosen as the host for blind passaging of N30 to select for gain-of-function mutants. After five serial passages, a mutant phage N30-5 was isolated. This mutant exhibited a significantly increased EOP on *E. coli* 94 compared with phage N30 (Table 1). However, the EOP of N30-5 remained significantly lower than that of C5. Further comparisons of other infection characteristics between N30-5 and N30 revealed no significant differences in plaque size (Fig. 1B through F), adsorption rate (Table 1), and growth curve (Fig. 1G through I). Interestingly, N30-5 exhibited a weaker bacteriostatic effect on *E. coli* 94 than N30 (Fig. 1K). Furthermore, amino acid sequence comparison between N30 and N30-5 proteins revealed that the only mutation in N30-5 was a G38510A mutation in N30-5_ORF44 (Fig. 4A), resulting in an A135T mutation at the protein level. N30_ORF44 encodes an HNH endonuclease and, as noted above, was the only ORF that could not be paired between phages C5 and N30. Notably, the mutation site in the N30-5 genome is not located within the conserved HNH domain but lies closer to the C-terminus of the protein.

HNH protein has been reported in some bacteria or phages with variable similarity and is known to have endonuclease activity (13–16). Given that a single nucleotide mutation within N30_ORF44, which encodes an HNH endonuclease domain, was sufficient to alter the EOP of phage N30 on *E. coli* 94, we further investigated the sequence variability of HNH-like sequences within the *Teseptimavirus* genus. Phages from the *Teseptimavirus* genus listed in the ICTV Master Species List 2023 were selected for comparison. Several phages were randomly selected from the BLASTp results, with varying coverage and identity to N30_ORF44 and C5_ORF28 (Table 2). Surprisingly, we found that sequences with high coverage (≥90%) to N30_ORF44 were all found adjacent to the genes coding for the terminase large subunit, which were located at the end of the phage genome. However, genes adjacent to sequences with high coverage of C5_ORF28 encoded a virion assembly protein, located in the middle of the genome. Among the *Teseptimavirus* phages listed in the ICTV Master Species List 2023, five phages showed no detectable HNH homologs with high similarity to C5_ORF28 or N30_ORF44 (Table S1). Notably, *Escherichia* phage HZ2R8 is the only *Teseptimavirus* phage that aligns with both C5_ORF28 and N30_ORF44 (100% coverage, >95% similarity), suggesting potential functional redundancy or divergence of the two HNH homologs within this phage.

**TABLE 2:** BLASTp analysis for amino acid sequences comparing *Teseptimavirus* phages to C5_ORF28 and N30_ORF44

Scientific name	Coverage (%)	Identity (%)	Length (aa)	Ranking/number of ORFs	Predicted function of adjacent proteins	Accession number
*Escherichia* phage C5	100.00	100	130	28/44	Virion assembly protein	AYD80198.1
*Escherichia* phage HZ2R8	97.71	100	130	22/38	Virion assembly protein	YP_009798029.1
*Escherichia* phage T7	71.76	100	130	28/60	Virion assembly protein	CAA24424.1
*Yersinia* phage YpP-Y	70.99	100	130	29/46	Virion assembly protein	AFK13373.1
*Yersinia* phage YpsP-G	70.99	100	130	34/52	Virion assembly protein	AFK13521.1
Enterobacteria phage 13a	49.24	100	131	36/56	Virion assembly protein	ACF15918.1
*Escherichia* phage N30	100.00	100	147	44/44	Terminase large subunit	YP_009813810.1
Enterobacteria phage vB_EcoP_IME390	95.95	100	147	45/46	Terminase large subunit	AYD82961.1
*Escherichia* phage HZ2R8	95.27	100	147	38/38	Terminase large subunit	YP_009798007.1
*Escherichia* phage CICC 80001	95.27	100	147	49/49	Terminase large subunit	AIM41056.1
*Escherichia* phage HZP2	94.60	100	147	42/43	Terminase large subunit	YP_009820198.1
*Salmonella* phage 3A_8767	93.92	100	146	48/48	Terminase large subunit	AXC37065.1

Given the divergence in adjacent functional proteins, we hypothesize that these two HNH endonucleases might exhibit functional differentiation in the phage, prompting us to analyze their sequence and structural characteristics. BLASTp analysis revealed that they share 54% coverage and 46% sequence identity, and Pfam predictions showed both proteins possess conserved HNH endonuclease domain belonging to the Clan His-Me_finger (Fig. 4A). Three-dimensional structure predictions further revealed substantial overlap between the N-terminal regions of the two proteins (Fig. 4B), extending beyond the conserved domain, indicating their tertiary structures are more conserved than their primary sequences. Interestingly, N30_ORF44 is one amino acid longer than C5_ORF28, with an extra residue at position S45 in N30_ORF44 (Fig. 4A and B), which may contribute to functional divergence between the two HNH endonucleases. Three-dimensional structural comparison yielded a root mean square deviation of 2.21 Å and a template modeling (TM) score of 0.53, indicating moderate structural similarity, with a 43% identity across the entire aligned region. To identify the potential catalytic residues of these HNH endonucleases, we performed ligand-binding site prediction using the COACH server. For C5_ORF28, the top-ranked predicted binding site (*C*-score = 0.57, cluster size = 42) involved three residues, C50, C55, and H60, which were predicted to coordinate a zinc ion through a Cys-Cys-His motif. This zinc-binding motif is characteristic of HNH endonuclease domains, where zinc ions typically stabilize the ββα-metal fold essential for catalytic activity. H60 corresponds to the conserved histidine within the HNH catalytic triad, which serves as the catalytic nucleophile critical for DNA cleavage. Notably, the distinct adjacent proteins flanking these HNH endonucleases in their respective genomes may contribute to functional divergence in virion assembly or DNA packaging, thereby influencing progeny production. These findings suggest that while the two HNH endonucleases share a partially conserved structural framework, they still differ evidently and likely perform distinct roles.

## DISCUSSION

Although phage C5 exhibited larger plaque sizes, higher adsorption rates, and greater EOP values across multiple bacteria, its overall bactericidal performance was unexpectedly weaker than that of phage N30. Several mechanisms may account for these contrasting outcomes. First, plaque size and EOP are strongly influenced by factors such as diffusion rate and latent period, which may not reflect phage replication dynamics in broth culture (17). Second, while C5 displayed higher adsorption rates, excessively rapid or high-frequency adsorption can lead to phenomena such as lysis without or reduced spatial dispersal of progeny phages, ultimately limiting productive infection (18). Third, the stronger inhibitory activity of N30 correlated with its larger burst size and higher progeny yield, suggesting that intracellular replication efficiency rather than initial adsorption may play a more decisive role in determining bactericidal capacity. For example, under identical culture conditions, phage T7 exhibits a lower adsorption efficiency than T4 or T5, yet still demonstrates high virulence, suggesting that a shorter latent period or larger burst size can compensate for reduced adsorption efficiency (19). Together, these findings highlight the complex interplay between phage-host interactions and emphasize that no single indicator, such as plaque size or EOP, is sufficient to evaluate therapeutic potential. Instead, comprehensive assessment of adsorption dynamics, burst size, and bactericidal activity is essential for selecting effective phages for clinical or biotechnological applications.

Our study provides new insights into the genetic variability and functional relevance of HNH endonucleases within the genus *Teseptimavirus*. We demonstrated for the first time that HNH genes in this genus have two distinct structures and occupy two separate genomic positions: one located in the middle of the genome adjacent to genes encoding virion assembly proteins and the other positioned at the terminal region next to the gene coding for the terminase large subunit. It is reported that in many long-tailed phages, genes encoding HNH endonucleases are frequently associated with terminase genes, and HNH proteins near terminases play a specific role in DNA packaging (14, 15). Our results extend this principle to T7-like short-tailed podoviruses, suggesting that the dual distribution of HNH is a conserved but previously underappreciated feature in *Teseptimavirus*. The occurrence of HNH endonucleases in two distinct genomic contexts and 3D structures suggests functional divergence. The HNH encoded in the middle region of the genome, such as C5_ORF28, is likely to participate in virion morphogenesis by assisting in the assembly of the capsid, tail, and other structural components, thereby contributing to the formation of complete viral particles. In contrast, the HNH encoded at the genome terminus, such as N30_ORF44, appears to be primarily involved in facilitating DNA packaging into pre-assembled capsids. Nevertheless, HNH endonuclease in modulating phage infection efficiency remains to be further substantiated, and its association with changes in EOP has not been directly validated. Therefore, alternative explanations cannot be excluded, including genetic linkage with additional mutations elsewhere in the genome, as well as epistatic interactions between the HNH endonuclease and structural or replication-associated proteins. In addition, host-related factors, such as dynamic changes in receptor expression and intracellular restriction-modification systems, may also influence phage infectivity. The observation that the A135T substitution significantly increased EOP on *E. coli* 94 but not on other hosts (Table 1) suggests that the effect of this HNH mutation is host-dependent. This host specificity raises the possibility that *E. coli* 94 harbors specific intracellular defenses that differentially interact with the HNH endonuclease during DNA packaging or replication. Alternatively, the HNH-mediated DNA cleavage may be particularly critical for productive infection in this host background. Regardless, these findings indicate that the molecular mechanism linking HNH to EOP may vary across bacterial hosts, and further investigation is needed to elucidate the underlying host-dependent factors.

Moreover, we observed that the gene pair C5_ORF40 and N30_ORF39, which share less than 50% sequence similarity, also exhibits marked variability across the *Teseptimavirus*. Although their classification within the infection-associated genomic module suggests a potential role in modulating infection dynamics, whether these genes directly affect phage infection remains unclear. The functional differences associated with HNH endonucleases and the variability between C5_ORF40 and N30_ORF39 likely underlie the phenotypic variations observed between the two phages, reflecting evolutionary divergence driven by adaptation to distinct bacterial hosts. While it is not possible to determine which phage strain possesses a higher infection efficiency, both strains may represent optimal evolutionary solutions fine-tuned to their respective hosts and ecological contexts.

## MATERIALS AND METHODS

### Bacterial strains and phages

Polyvalent phages N30 and C5 were isolated from two pig farms, respectively (4). Their five bacterial host strains are C. *freundii* 113ju, *E. coli* 94, *E. coli* 121, *E. coli* 141, and *E. coli* 235, all isolated from pig stool samples previously. *C. freundii* 113ju was used as the host for phage cultivation.

### Transmission electron microscopy

Phage supernatant (10^10^–10^11^ PFU/mL) was applied to carbon-coated copper grids for 15 min. After staining with 2% phosphotungstic acid (pH 7.2), phage particles were observed using a transmission electron microscope (HT7700, HITACHI, Tokyo, Japan) at 80 kV.

### Plaque morphology observation

Plaque morphology was assessed using double-layer agar methods for each phage on different bacteria. Plaque images were captured from the agar plates after incubation. For quantitative analysis, randomly selected isolated plaques (at least 12 per plate) were measured for diameter using calibrated image software.

### Determination of efficiency of plating

The efficiency of plating was measured as described previously (9). Briefly, the same number of phages was incubated with each strain in Luria-Bertani (LB) double-layer agar at 37°C for 6 h. The number of plaques on *C. freundii* 113ju was considered as the reference (EOP = 1), and EOP values were calculated as the ratio of the number of plaques on each strain to the number of plaques on *C. freundii* 113ju. Each experiment was repeated at least three times with three replicates.

### Determination of phage adsorption rate

All bacterial strains were grown in LB medium overnight at 37°C before adsorption. Equal volumes of phages were mixed with each bacterium at a multiplicity of infection (MOI) of 0.01 in 1 mL of LB broth, followed by incubation at 37°C for 10 min. The mixture was centrifuged at 4,000 rpm for 5 min, and the supernatant containing free phages was discarded. The pellet was resuspended twice with fresh phosphate-buffered saline to remove free phages. After serial dilution, the titer of adsorbed phages was determined using the double-layer agar plate method. The phage adsorption rate was calculated as the ratio of adsorbed phage titer to the initial phage titer added. Each experiment was repeated at least three times with three replicates.

### Bacteriostatic assay and one-step growth curve

Bacteriostatic assays were performed in *E. coli* 94, *E. coli* 235, and *C. freundii* 113ju to evaluate the effect of phages on bacterial growth. Briefly, phages and bacteria were mixed at an MOI of 0.01 and incubated at 37°C. Bacterial growth in the presence of phages was monitored by measuring optical density at 600 nm (OD600) every hour for 7 h. LB medium inoculated with bacteria only was used as the control. For one-step growth curves, phages and bacteria were mixed at an MOI of 0.01 and incubated at 37°C for 10 min. The mixtures were centrifuged at 4,000 rpm for 5 min to remove unadsorbed phages, and the pellets were resuspended in fresh LB medium and incubated at 37°C. Phage growth was monitored by collecting samples at different time points over 300 min, followed by serial dilution and phage titer determination using the double-layer agar method. The latent period was defined as the interval before detectable phage release, the burst period as the phase of rapid phage release, the burst size as the ratio of released phages to infected bacterial cells, and the peak titer as the maximum phage concentration reached during the assay (10). Each experiment was repeated at least three times with three replicates.

### Phage genome sequencing, annotation, and comparative analysis

Phage genomic DNA was extracted according to a protocol from *Molecular Cloning: A Laboratory Manual* (20). Briefly, phage particles were precipitated with 10% PEG 8000 and 0.5 M NaCl, followed by proteinase K and SDS lysis. The released DNA was purified by phenol-chloroform extraction, ethanol-precipitated, and dissolved in nuclease-free water. The extracted DNA was used for whole-genome sequencing at Biorezon using an Illumina HiSeq paired-end platform. Raw reads were quality-filtered using Trimmomatic v0.33 (21), and *de novo* genome assembly was conducted using SPAdes v3.13.11 (22). Terminal repeats were identified by pairwise alignment of the 5′ and 3′ genome termini using BioEdit and confirmed by elevated sequencing coverage at both ends. Open reading frames (ORFs) were predicted by GeneMarkS (23). Comparative genome analysis of phages C5 and N30 was performed using BLASTp against databases, with an *E*-value threshold of ≤1 × 10^−5^. Pairwise genome comparison was visualized using Clinker v0.0.32 (24).

### Protein sequence alignment and 3D structure prediction

For N30_ORF39 and C5_ORF40, homologous proteins identified within members of the subfamily *Studiervirinae* were retrieved, and the best BLASTp hit from each genus was selected for comparison. Amino acid identity of the best BLASTp hits was visualized as heatmaps using the ggplot2 package in R Studio (25). For N30_ORF44 and C5_ORF28, all classified *Teseptimavirus* phages listed in the ICTV Master Species List 2023 were selected for comparative analysis. Homologous regions identified by BLASTp were annotated. For HNH endonuclease analysis, amino acid sequences of N30_ORF44, N30-5_ORF44, and C5_ORF28 were aligned using ClustalW Omega (26). Conserved domains were identified through the Pfam database (27). Then, the three-dimensional structure of the ORFs containing an HNH endonuclease domain was predicted with AlphaFold2 (28), and a structural alignment against RCSB PDB templates was conducted using TM-align with default parameters (29). Putative ligand-binding and catalytic sites were predicted using the COACH server (30).

### Screen for mutants

Phage N30 was propagated by blind passaging on *E. coli* 94 for five successive rounds. Briefly, at each round, phage lysate was added to a fresh exponential culture of the host strain, incubated until lysis, and filtered. After the fifth passage, 50 plaques were randomly picked and purified individually for EOP determination on *E. coli* 94. Putative mutant phage showing significantly increased EOP was selected for whole-genome sequencing, and the mutant strain was designated as N30-5.

### Statistical analysis

Statistical analysis was performed using SPSS v25.0.0.2 and GraphPad Prism v9.4.1. One-way analysis of variance was used for experiments with multiple variables, and multiple comparisons were carried out using Duncan. Comparisons with *P* < 0.05 were considered significant.

## Data Availability

The genome sequences of phages N30 and C5 were deposited in GenBank under accession numbers MH717098 and MH717099, respectively. The GenBank accession numbers for the bacterial host strains are as follows: *C. freundii* 113ju, MH643693; *E. coli* 94, MH671448; *E. coli* 121, MH671455; *E. coli* 141, MH671461; and *E. coli* 235, MH671490.

## SUPPLEMENTAL MATERIAL

Table S1 aem.01347-26-s0001.xlsx BLASTp analysis of C5_ORF28 and N30_ORF44 against all *Teseptimavirus* phages listed in the ICTV Master Species List 2023.

ASM does not own the copyrights to Supplemental Material that may be linked to, or accessed through, an article. The authors have granted ASM a non-exclusive, world-wide license to publish the Supplemental Material files. Please contact the corresponding author directly for reuse.
